# Interventions to increase uptake of cervical screening in sub-Saharan Africa: a scoping review using the integrated behavioral model

**DOI:** 10.1186/s12889-020-08777-4

**Published:** 2020-05-11

**Authors:** Breanne E. Lott, Mario J. Trejo, Christina Baum, D. Jean McClelland, Prajakta Adsul, Purnima Madhivanan, Scott Carvajal, Kacey Ernst, John Ehiri

**Affiliations:** 1 Mel & Enid Zuckerman College of Public Health, University of Arizona, 1295 N. Martin Ave., Tucson, AZ 85724 USA; 2 Health Sciences Library, University of Arizona, 1501 N Campbell Ave Ward 6, Tucson, AZ 85724 USA; 3 School of Medicine and Cancer Center, University of New Mexico, MSC08 4720, 1 University of New Mexico, Albuquerque, NM 87131-0001 USA; 4 College of Medicine, University of Arizona, 1501 N Campbell Ave, Tucson, AZ 85724 USA; 5 Public Health Research Institute of India, 89/B, 2nd Cross, 2nd Main, Yadavgiri, Mysore, 560021 India

**Keywords:** Cervical cancer, Screening, Secondary prevention, Review, Sub-Saharan Africa, Health behavior

## Abstract

**Background:**

Sub-Saharan Africa (SSA) experiences disproportionate burden of cervical cancer incidence and mortality due in part to low uptake of cervical screening, a strategy for prevention and down-staging of cervical cancer. This scoping review identifies studies of interventions to increase uptake of cervical screening among women in the region and uses the Integrated Behavioral Model (IBM) to describe how interventions might work.

**Methods:**

A systematic search of literature was conducted in PubMed, Web of Science, Embase, and CINAHL databases through May 2019. Screening and data charting were performed by two independent reviewers. Intervention studies measuring changes to uptake in screening among women in SSA were included, with no restriction to intervention type, study setting or date, or participant characteristics. Intervention type and implementation strategies were described using behavioral constructs from the IBM.

**Results:**

Of the 3704 citations the search produced, 19 studies were selected for inclusion. Most studies were published between 2014 and 2019 (78.9%) and were set in Nigeria (47.4%) and South Africa (26.3%). Studies most often assessed screening with Pap smears (31.6%) and measured uptake as ever screened (42.1%) or screened during the study period (36.8%). Education-based interventions were most common (57.9%) and the IBM construct of knowledge/skills to perform screening was targeted most frequently (68.4%). Willingness to screen was high, before and after intervention. Screening coverage ranged from 1.7 to 99.2% post-intervention, with six studies (31.6%) reporting a significant improvement in screening that achieved ≥60% coverage.

**Conclusions:**

Educational interventions were largely ineffective, except those that utilized peer or community health educators and mHealth implementation strategies. Two economic incentivization interventions were moderately effective, by acting on participants’ instrumental attitudes, but resulted in screening coverage less than 20%. Innovative service delivery, including community-based self-sampling, acted on environmental constraints, striving to make services more available, accessible, and appropriate to women, and were the most effective. This review demonstrates that intent to perform screening may not be the major determinant of screening behavior, suggesting other theoretical frameworks may be needed to more fully understand uptake of cervical screening in sub-Saharan Africa, particularly for health systems change interventions.

## Background

Cervical cancer is among the most common cancers worldwide and disproportionately affects African women [1]. As Africa experiences an epidemiologic transition, with aging populations that are susceptible to lifestyle diseases, the continent accounts for an increasing proportion of global cancer cases and deaths. Regional variations in cervical cancer are especially marked; Sub-Saharan Africa has the highest rates of cervical cancer in the world and cervical cancer is the number one cancer-related cause of mortality in the region [2]. The age-standardized mortality rate in East Africa is 30.0 per 100,000 compared to just 1.9 per 100,000 in North America, almost a 16-fold higher rate [2]. Incidence, similarly, is six times higher in East Africa than North America (40.1/100,000 vs. 6.4/100,000) [2].

Most cervical cancer is caused by Human Papillomavirus (HPV) infection, a common and often asymptomatic sexually transmitted infection. As a disease with both infectious and non-infectious etiologic components and risk factors, such as an increased risk for cervical cancer among women living with HIV, the cervical cancer epidemic in Africa is both profound and complex. The African cervical cancer epidemic is characterized by the double burden of communicable and non-communicable disease [3], human resource for health shortages [4], preventive health service delivery challenges [5–7], lack of access to treatment, and low cervical cancer awareness among the population and health providers [8, 9].

Since cervical cancer is a preventable cancer, screening is an important cancer control and prevention strategy, recommended by the World Health Organization (WHO) for all women aged 30 years and older, and beginning even earlier for some high-risk groups such as women living with HIV [10]. Many African countries have adopted a “screen-and-treat” approach in recent years, aimed at employing routine screening of asymptomatic adult women for early detection and on-the-spot treatment of cervical pre-cancerous lesions. Due to resource and infrastructure constraints, cytology-based screening methods like Pap smears may not be feasible for some African communities or may cause service delivery bottlenecks that prevent screening programs from achieving high coverage. Alternatively, visual inspection of the cervix with acetic acid (VIA) or Lugol’s iodine (VILI) may provide a low-cost screening alternative that can be implemented by various cadres of trained health workers [5, 6, 10]. Additional screening methodologies include HPV testing, which can utilize provider-collected or patient-collected sampling techniques. Still, uptake of screening services remains low in Africa [6], with women citing barriers of fear of the procedure and outcomes, cultural issues such as stigmatization, breach of modesty, inaccessibility of screening services, and a view that screening services are unnecessary if one is feeling well [8].

The aim of this scoping review is to map the literature on interventions to increase uptake of cervical screening in sub-Saharan Africa and identify opportunities for future intervention development and research. The Integrated Behavioral Model (IBM) is applied as a health behavior framework to understand how interventions might work to improve screening behavior.

## Methods

This scoping review was conducted from April to October 2019, utilizing a rigorous systematic search of four electronic publication databases, a structured two-step screening process conducted by multiple independent reviewers, and an iterative data charting process that employed the IBM to describe the nature of interventions conducted to increase cervical cancer screening among women in sub-Saharan Africa. Given the wide range in approaches to increasing cervical cancer screening, the many different screening methods being practiced and evaluated, and the goal of identifying gaps in knowledge through mapping to a behavior change model, we determined scoping methodology was best suited for this review [11, 12]. Findings are reported according to Preferred Reporting Items for Systematic reviews and Meta-Analyses extension for Scoping Reviews (PRISMA-ScR) Checklist [13].

### Search and screening

A medical librarian (JMC) was consulted to develop a comprehensive search strategy, to identify appropriate databases, and to conduct the literature search. We systematically searched electronic databases PubMed/MEDLINE (1946-May 1, 2019), Clarivate Analytics Web of Science (1900-May 1, 2019), Elsevier EMBASE (1947-May 1, 2019), and EBSCO CINAHL (1982-May 1, 2019). The searches utilized controlled vocabulary searching modified for each database, as well as natural or key terms following a structure of “topic/cervical cancer” AND “outcome/screening uptake” AND “context/population.” No language or publication date restrictions were applied. Additional file 1: Appendix 1 details the complete search strategy. Conference proceedings and abstracts, published as supplements to journals or indexed in the electronic databases, were returned in the search and were screened with the same eligibility criteria as peer-reviewed articles.

The search results were compiled and deduplicated using EndNote (version × 8). Then, the deduplicated entries were uploaded to Covidence, an online software for systematic screening of results. Screening was completed in two rounds: title and abstract screening was performed, then full text review for eligibility. Each search result was screened independently by two reviewers (BEL, MJT, CB). If the two reviewers were not in agreement, the result was screened by a third reviewer (BEL, MJT, CB).

### Eligibility criteria

Inclusion criteria were studies with an intervention component, conducted in a sub-Saharan African community, and any type of participant such as women, their male partners or other family members, or health care providers. Studies were excluded if they targeted African participants in a non-African setting as were studies measuring attendance of follow-up care for women who had previously attended cervical cancer screening. All intervention types were considered including one-on-one or group educational interventions or counseling, patient reminders, media and awareness-building campaigns, incentivization schemes, and innovative service delivery approaches, which could be set in health facility- or community-based contexts. The primary outcome of interest was uptake of cervical screening, measured as self-reported or medically verified receipt of any type of cervical cancer screening service, as a proportion of the entire study population. Secondary outcomes of interest were cervical cancer or screening knowledge/awareness, willingness/intent to get screened, and cervical cancer or screening-related attitudes/beliefs. Comparisons varied and included no intervention and standard care. Study designs that could demonstrate a change in screening uptake over time or between two groups were included: randomized control trials, observational cohort studies, and before-and-after studies. Studies that described screening program implementation, without screening rates for a comparison group were excluded.

### Data charting and analysis

An excel data charting form, created and tested by our team, was used to elicit information on pre-defined variables from the included sources. The form was modified during an iterative data charting process. Data extracted included: study period, location, sample size, aims of the study, methodology, participants/population, intervention, comparison, findings, and author recommendations. In addition to descriptions of the intervention, information was recorded about the intervention development process and the delivery. Data charting for each included study was completed by two reviewers (BEL, MJT, CB). Reviewers used emergent themes to categorize the studies by intervention type. Content analysis was performed, using the IBM as a framework to describe the scope of interventions included, as described in the following section. Each study was reviewed for evidence that the researchers considered IBM constructs during the development of their intervention, as the mechanism through which the intervention is supposed to act, or as a measure or outcome of the intervention study. Presence or absence of IBM constructs was recorded for each study and the compiled construct frequency data is presented in a table. Narrative synthesis is used to elaborate on ways in which the constructs appeared in the included studies. An optional quality assessment of the included studies was not performed, as it is unnecessary for scoping review methodology and did not help us to meet our study objectives [11].

### Integrated behavioral model

**Fig. 1:**
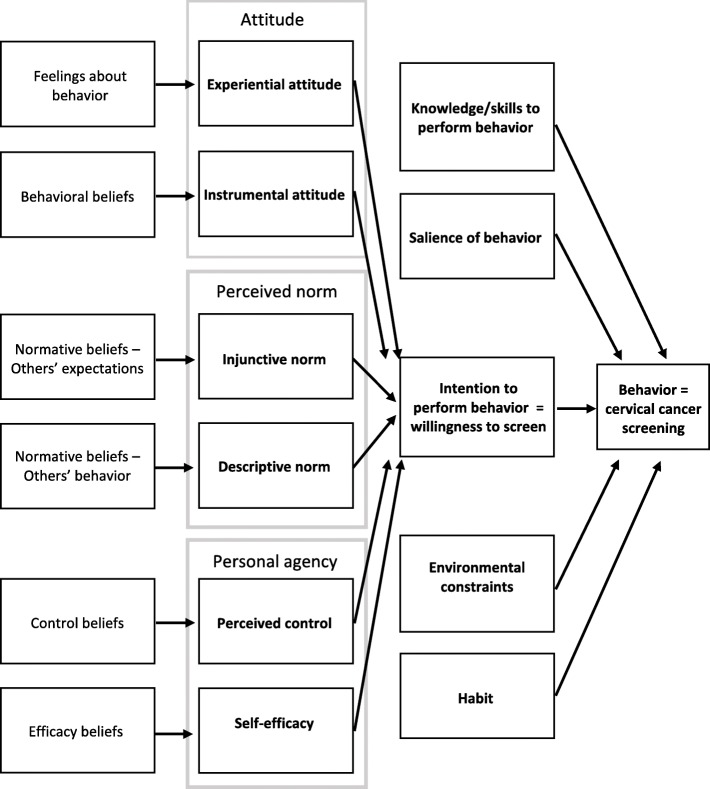
The Integrated Behavioral Model (IBM), adapted for cervical cancer screening behavior

As the purpose of this review is to describe the nature of interventions that have been conducted to increase uptake of cervical cancer, we chose to use a health behavior model to map the breadth of literature, to understand how the interventions might work to create behavior change, and to identify gaps in intervention types and implementation strategies that have been attempted. The IBM was developed by bringing together constructs from the Theory of Planned Behavior, the Theory of Reasoned Action, and other prominent health behavior theories with overlapping constructs [14]. The main premise of the IBM is that the most important determinant of a behavior is intention to perform that behavior, and that intention is influenced by attitudes, perceived norms, and personal agency [14]. Factors such as knowledge and environmental constraints also act on the behavior directly (see Fig. 1). This review uses the IBM to understand cervical screening as a behavior, “willingness to screen” as the precursive intention to perform the screening behavior, and other theoretical constructs to describe the rationale, action, and evaluation of interventions intended to change screening behavior.

## Results

### Study characteristics

**Fig. 2:**
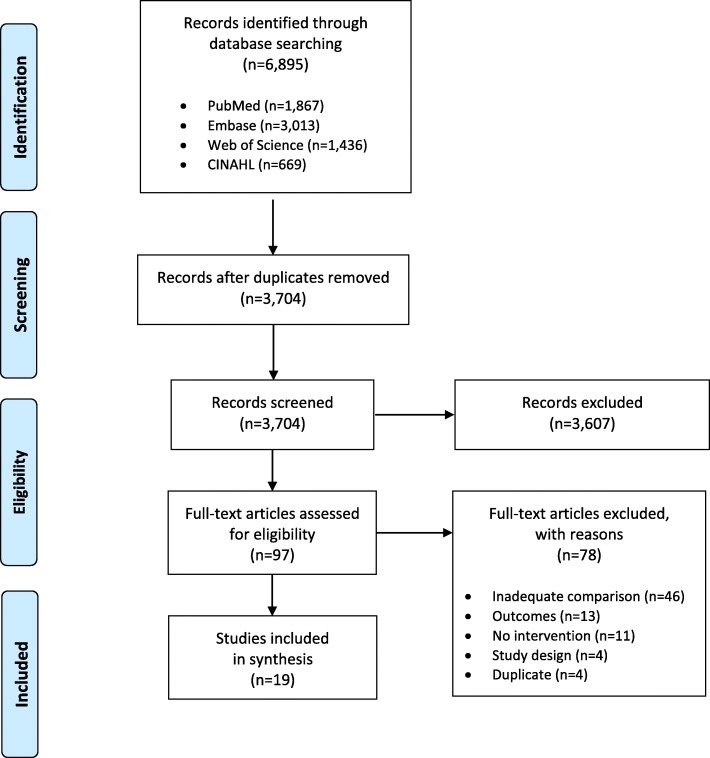
Flow diagram of source selection process

The systematic search returned 3704 unique entries, after duplicates were removed. Screening and application of eligibility criteria produced 19 studies for inclusion in the review [15–33]. Figure 2 shows the results of the study selection process. The majority of studies were published between 2014 and 2019 (*n* = 15, 78.9%), and were randomized (*n* = 9, 47.4%) or quasi-experimental studies (*n* = 5, 26.3%). Studies were set in seven African countries with almost half set in Nigeria (n = 9, 42.9%), followed by South Africa (n = 5, 23.8%) and Kenya (*n* = 3, 14.3%). While not all studies specified, both urban and rural settings were represented. Minimum age eligibility criteria were used to define target populations; few studies cited World Health Organization or other screening recommendations when defining age criteria. Pap smear or cytology-based cervical screening was assessed most often (*n* = 6, 31.6%) [16, 17, 21, 26, 31, 33]. A quarter of studies (*n* = 5, 26.3%) did not specify which screening test was being performed [20, 22, 24, 29, 32]. Multiple screening methods were used in some studies, such as those that compared uptake of HPV self-sampling to facility-based visual inspection screening methods [19, 25, 27, 28]. Screening was offered at workplaces [28], in women’s homes [19, 27, 28], in community spaces [23], and in health facilities [15–18, 20–22, 24–26, 29–33]. Study characteristics are presented in Table 1.

**Table 1:** Descriptive statistics of study period, context, and methodology of included studies

Study characteristic	Total (***n*** = 19)
	N (%)
Year of publication	
Before 2014	4 (21.1%)
2014–2016	6 (31.6%)
2017–2019	9 (47.4%)
Study design	
Randomized/cluster-randomized	9 (47.4%)
Quasi-experimental	5 (26.3%)
Before-and-after	4 (21.1%)
Other non-randomized	1 (5.3%)
Country (*n* = 21)	
Nigeria	9 (42.9%)
South Africa	5 (23.8%)
Kenya	3 (14.3%)
Tanzania	1 (4.8%)
Uganda	1 (4.8%)
Mozambique	1 (4.8%)
Zambia	1 (4.8%)
Urban/rural setting	
Urban/semi-urban only	6 (31.6%)
Rural only	4 (21.1%)
Both urban and rural	1 (5.3%)
No information	8 (42.1%)
Sample size	
< 250	2 (10.5%)
250–499	4 (21.1%)
500–749	4 (21.1%)
750–1000	3 (15.8%)
> 1000	6 (31.6%)
Screening method	
Pap smear/cytology-based	6 (31.6%)
Visual inspection	3 (15.8%)
HPV DNA testing	1 (5.3%)
Multiple methods	4 (21.1%)
No information	5 (26.3%)
Minimum eligible screening age, in years	
< 18	1 (5.3%)
18–24	5 (26.3%)
25–29	3 (15.8%)
30–34	4 (21.1%)
35+	1 (5.3%)
No information	5 (26.3%)

We found three emergent themes for intervention types: health education interventions (*n* = 11, 57.9%), economic incentivization interventions (*n* = 2, 10.5%), and innovative service delivery interventions (*n* = 6, 31.6%). Descriptions of the population, intervention, comparison, and outcomes (PICO) of included studies, organized by intervention type, can be found in Table 2. The most prevalent type of intervention was health education which was characterized by use of short-term, lecture-based education, peer health educators, multimedia lessons, and behavior change communication and message framing interventions. Educational interventions operated under the rationale that low knowledge and awareness of cervical cancer and screening services are the primary barriers to screening uptake. These interventions were implemented in-person and remotely, through email and text messaging. There were two economic incentivization interventions, which included a lottery-style game that was played by women to randomize their households to receive subsidized cervical screening and cancer treatment in the unlikely event that cancer was detected and a reward program offered by a health insurance plan that incentivized receipt of preventive health services including cervical screening, where women could earn points and prizes for their behavior [26, 30]. Six of the included studies described innovative approaches to delivering cervical screening including: changes to the type of cervical screening provided, the screening location, or the coupling of cervical screening with other services with the intent to make cervical screening more comfortable, convenient, and accessible by removing or mitigating environmental barriers to screening. Cervical screening was integrated with sexual and reproductive health services in two studies [22, 24], and with HPV vaccination for adolescent daughters in a school-based intervention [19]. Multiple studies also drew upon established social ties; interventions targeted male partners [22] and peer groups of female sex workers [24], utilized trusted community health outreach workers and religious leaders to deliver interventions [18, 25, 28], and leveraged the influence of the King’s council in one community [27].

**Table 2:** Description of interventions to increase uptake of cervical screening in sub-Saharan Africa and related outcomes

Study	Population	Intervention(s) and Comparison(s)	Outcomes
**Health Education Interventions**			
Abiodun 2014 [15]	Women, aged 25–64, in rural Nigeria	a. Multi-component structured health education on cervical cancer and prevention featuring a culturally-relevant home video, didactic lectures, discussion session, and informational pamphlet delivered during one 4-h session. b. Health education on breast cancer without a culturally-relevant video.	Follow-up: 13 weeks Uptake: 4% difference in “ever screened” pre- to post-intervention (4.3 to 8.3%, *p* = 0.038) in intervention vs. 0.4% difference in control. Awareness: 83.1% difference in “ever heard of cervical cancer” pre- to post-intervention (16.9 to 100%, *p* < 0.0001) in intervention vs. 1.3% difference in control. An 89.7% difference in “ever heard of screening” (10.3 to 100%) in intervention vs. 0.2% difference in control. Knowledge: 68.5% difference in “very good” knowledge from pre- to post-intervention (2 to 70.5%, *p* < 0.0001); mean knowledge score increased 23.94 points (out of 40, 1.75 to 25.69) in intervention vs. 0.19 score increase in control. Willingness to screen: Non-significant difference from pre- to post-intervention (89.7 to 92.3%, *p* = 0.283) in intervention vs. 2.0% difference in control.
Adamu 2012 [16]	Female teachers in secondary schools, in Nigeria	a. Health education on cervical cancer and prevention, with an emphasis on cervical cytology using Pap smear, delivered via lecture presented twice (1 month apart) and a Pap smear demonstration. Each participant received a coupon for a free Pap smear test. b. No intervention (delayed intervention).	Follow-up: 3 months Uptake: No significant increase in uptake from pre- to post-intervention in intervention group (1.1 to 3.4%, *p* = 0.45) or control. Attitude: Mean attitude score improved by 17.4 points (35.4 to 52.8, *p* < 0.001) in intervention vs. 1.9 point decrease in control. Knowledge: Mean knowledge of cervical cancer score increased by 31.7 points (25.5 to 57.2, p < 0.0001) in intervention vs. 11.5 points in control. Mean knowledge of Pap smear score increased by 10.9 points (17.1 to 28.0, p < 0.0001) in intervention vs. 1.3 point decrease in control.
Adonis 2017 [17]	Health-insured females, aged 21–65, in South Africa	a.i. A structured email using loss-framed messaging about cervical cancer and screening plus a request to attend screening, and one reminder email 3 months later. Messaging focused on risk with phrases like “too late” and “bad health outcomes”. a.ii. A structured email using gain-framed messaging about cervical cancer and screening plus a request to attend screening, and one reminder email 3 months later. Messaging focused on improving health and well-being with phrases like “better health”. b. A structured email using neutrally-framed messaging to provide only factual statements on recommendations for screening plus a request to attend screening, and one reminder email 3 months later.	Follow-up: 6 months after first message Uptake: No statistically significant difference between the screening rates of the groups during the study period (8.81% in loss-framed intervention, 5.71% in in gain-framed intervention, 9.58% in control, *p* = 0.75). Measure of “ever screened” not reported.
Chigbu 2017 [18]	Women, aged 30 and older, in rural Nigeria	a. House-to-house cervical and breast cancer prevention education, delivered one-on-one using a structured information booklet, by a trained community health educator. Screening services were free, and HPV vaccination was offered for eligible children for a fee. Facilities were within walking distance in each community. b. Before-and-after study design; each participant is their own control	Follow-up: 6 months Uptake: 64.4% difference in “ever screened” from pre-to post-intervention (3.2 to 67.6%, *p* < 0.001). Awareness: Of the women screened after the intervention, 94.3% were not aware of screening before the intervention.
Erwin 2019 [20]	Women, aged 25–49, in urban and rural Tanzania	a.i. An SMS behavior change communication intervention, with a series of 15 text messages about cervical cancer and context-specific barriers to screening, delivered over 21 days. a.ii. The same SMS behavior change communication series + an eVoucher to cover transportation costs to the screening clinic, and a reminder message when the voucher was set to expire. b. One SMS message (sent up to three times) with the location and hours of the closest screening clinic.	Follow-up: 2 months Uptake: 8.6% difference in screening during the follow-up period in SMS only intervention group compared to control group (12.9% vs. 4.3%; AOR = 3.0, 95% CI: 1.5–6.2); 13.7% difference in SMS + eVoucher intervention group than control group (18% vs. 4.3%; AOR = 4.7, 95% CI: 2.93–7.44). Measure for “ever screened” not reported.
Gana 2017 [21]	Women, registered as venders with local market association, in Nigeria	a. Health education on cervical cancer delivered through two sessions held 4 weeks apart, with Information, Education, and Communication (IEC) materials distributed at the end of each session. b. No intervention (delayed intervention).	Follow-up: 3 months after second session Uptake: No significant change in “ever had Pap smear” from pre- to post-intervention (1.1 to 3.4%, *p* = 0.621). Awareness: 28.4% difference in awareness of Pap smear test between intervention and control (34.1% vs. 5.7%, *p* < 0.0001).
Mbachu 2017 [25]	Women, aged 21+, who worshipped in selected Anglican dioceses, in Nigeria	a. Health education on cervical cancer and prevention, delivered by peer educators (clergy wives) in 3 to 6 group sessions, 1–1.5 h each, over a period of 3 months. Sessions included didactic teaching and discussion. b. Before-and-after study design; each participant is their own control.	Follow-up: Unclear Uptake: 6.8% difference in “ever screened” from pre- to post-intervention (10.5 to 17.3%, *p* = 0.02). Willingness to screen: No significant increase from pre- to post-intervention (79.3 to 80.8%, *p* = 0.52). Other outcomes reported: perceived severity of cervical cancer, individual risk perception, perceived benefit of screening
Ndikom 2017 [29]	Women utilizing maternal health services, in Nigeria	a. Health education on cervical cancer and prevention, delivered by nurses through one group session, guided by standardized flex charts. Intervention sites were four health centers. b. No intervention, women attending antenatal clinics at control hospitals.	Follow-up: 6 months Uptake: No significant difference between intervention and control groups post-intervention (3.6% vs. 2.3%, *p* = 0.27). Awareness: 58.6% difference in “ever heard about cervical cancer” from pre- to post-intervention in intervention group (12.9 to 71.5%) vs. 3.9% difference in control (*p* = 0.001). 40.1% increase in awareness of where to get screened (12.9 to 53.0%) in intervention vs. 2.3% decrease in control (*p* < 0.001). Knowledge: 50.9% difference in proportion of participants with “poor” knowledge from pre- to post-intervention (94.2 to 43.3%) vs. 10% difference in “poor” knowledge in control (p < 0.001). Willingness to screen: 15.2% difference from pre-to-post-intervention (75.8 to 91.0%) in intervention group vs. 13.8% difference (71.5 to 85.3%) in control (*p* = 0.01).
Risi 2004 [31]	Women, aged 35–65, in peri-urban South Africa	a.i. Health education, utilizing a 20-page long photo-comic with information about cervical cancer and stages of cervical cancer, administered door-to-door. a.ii. Health education, in the format of a radio-drama, broadcast on a local radio station 10 times over 1 month. b.i. Photo-comic with educational information on personal finances and no health care messages. b.ii. Retrospective cohort design; comparison are participants who did not recall hearing the radio-drama.	Follow-up: 6 months Uptake: No significant difference in uptake of Pap smear during the study period between intervention and control groups (6.4% vs. 6.7%, *p* = 0.89). At baseline, 45% of women reported “ever had smear”. Of the 43 women who reported uptake, most (44.2%) did not recall the photo-comic or radio-drama; 25.6% recalled the photo-comic alone; 20.9% recalled the radio-drama alone; 11.8% recalled both. No evidence of interaction between the two interventions.
Rosser 2015 [32]	Women, aged 23+, in rural Kenya	a. A 30-min health education intervention consisting of an interactive talk about cervical cancer and guided discussion about barriers to screening and fears/stigma associated with screening. The intervention was guided by a flip-chart and script, and delivered by community health workers to groups of 4–6 women recruited from health facilities waiting areas. b. Standard of care; women were informed that screening was available, no health education.	Follow-up: 3 months Uptake: No significant difference in screening during the study period between intervention and control groups (58.9% vs. 60.9%, *p* = 0.60). Measure of “ever screened” not reported. Awareness: Mean awareness score increased by 1.4 points (2.6 to 4.0) in intervention vs. 0.9 points (2.4 to 3.3) in control (*p* < 0.01). Knowledge: Mean cervical cancer knowledge score increased by 2.3 points (8.7 to 11.0) in intervention vs. 1.5 points (8.5 to 10.0) in control (p < 0.01).
Wright 2011 [33]	Women, registered with the local market association, in urban Nigeria	a. Health education on cervical cancer screening tests, with emphasis on Pap smear, its benefits and procedure, and facilities where services are provided. The intervention included counseling and culturally-tailored and reader-friendly educational materials and was delivered during weekly market meeting periods. b. Health education on hypertension with blood pressure measurements provided.	Follow-up: 3 months Uptake: No significant difference in “ever had Pap smear test” from pre- to post-intervention in intervention (1.1 to 1.7%) or control group (*p* > 0.05). Awareness: 49.7% difference in awareness of Pap smear from pre- to post-intervention (6.9 to 56.6%) in intervention vs. 2.3% difference in control (*p* < 0.001). Willingness to screen: 19.5% difference from pre- to post-intervention (63.4 to 82.9%) in intervention vs. 3.4% increase in control (p < 0.01).
**Economic Incentive Interventions**			
Mehrotra 2014 [26]	Women, aged 16+, enrolled in a health plan and the plan’s optional paid incentive program, in South Africa	a. A preventive health incentivization program, offered by a health insurance plan, that offers rewards like movie tickets or international airfare, for receipt of 8 qualifying preventive health services including cervical screening. b. Plan members not enrolled in the incentivization scheme.	Follow-up: 1 year Uptake: The intervention group had increased odds of annual receipt of Pap smear, compared to the non-enrolled group (OR = 2.17, p < 0.01). The average annual receipt for the intervention group was 19.7%. Measure of “ever screened” not reported.
Okeke 2013 [30]	Females, aged 18–64, in Nigeria	a.i. Health education about cervical cancer and screening benefits + a subsidy to receive screening for 0 Naira (N), 50 N, or 100 N, as determined by playing a lottery scratch card game administered during a home-visit. a.ii. Same health education, as well as information about cancer prevalence + a cancer treatment subsidy to cover the cost of care *if* cancer was found during screening (up to N100,000), as determined by playing a lottery scratch card game administered during a home-visit. b. Health education only, without subsidy (those who “lost” the lottery game).	Follow-up: Unclear Uptake: 4.5% difference in intervention that received the treatment subsidy compared to control (17% vs. 12.5%). A N10 price increase of screening, reduced uptake by about 0.6 percentage points (N0: 18%, N50: 15%, N100: 11.2%).
**Innovative Service Delivery Interventions**			
Dreyer 2015 [19]	Mothers of girls in grades 4–7, who were in a school-based HPV vaccination program, in South Africa	a. A school-based educational intervention, consisting of a 15-min powerpoint presentation by a medical doctor and information leaflets, targeting school girls eligible for HPV vaccination and their parents. Mothers were invited to screen at a clinic or with a self-screening kit, based on study site. b. Before-and-after study design; each participant is their own control.	Follow-up: 6 months Uptake: 15.3% difference in “ever screened” from pre- to post-intervention (53.6 to 68.9%, *p* < 0.005). Screening scores were also assigned based on how long women were screened and if they had been screened more than once. The intervention was associated with significantly more recent screening. A more favorable change was observed for the self-collection intervention group; 45.3% of the self-collection group returned a screening kit. Knowledge: “Adequate” knowledge of cervical cancer increased 32.3% pre- to post-intervention (30.6 to 62.9%, p < 0.005).
Hewett 2016 [22]	Males and females, aged 18+, seeking select health services in catchment area, in urban/semi-urban Zambia	a.i. Enhanced client-centered counseling, referral to add-on services (including cervical screening) for the patient and their partner, with client follow-up by phone. A standardized assessment form, informational materials about the add-on services, and motivational interviewing were used at time of care seeking and 7 days later if the client failed to access referral services. a.ii. Same intervention + additional offer of immediate escort to the add-on service. Escorts guide the client at the time of referral, to the site of the add-on service. b. Standard of care for family planning, HIV testing and counseling, and voluntary medical male circumcision services. Normal client assessment and counseling, ad-hoc referrals (usually client-initiated), no direct transition or linkage between services, no follow-up of clients.	Follow-up: 6 weeks and 6 months Uptake: Significant increase in both interventions vs. control (*p* < 0.001; 22.2% uptake at 6 months vs. 9.7%, AOR 2.75, 95% CI: 1.94–3.91 intervention 1 vs. control; 23.6% vs. 9.7%, AOR = 2.98, 95% CI: 2.10–4.22 intervention 2 vs. control). There was little meaningful difference between the intervention arms, escort services are not very important for improving cervical screening uptake.
Huchko 2017 [23]	Women, aged 25–65, in rural Kenya	a. HPV screening was offered through periodic community health campaigns (CHCs), that utilized pop-up tents to offer community-based screening. Community health volunteers conducted community outreach and mobilization, screening, and result notification and feedback over a 6-week period, in select communities. b. Community outreach and educational information in control communities was the same, with offer of HPV self-testing, but community-based tents were not used. Instead, women were referred to their local government health facilities.	Follow-up: 6 weeks Uptake: 23% difference in screening during intervention period in intervention vs. control communities (60% vs. 37%, *p* < 0.001). Measure for “ever screened” not reported.
Lafort 2018 [24]	Female sex workers, aged 30+, in South Africa, Mozambique, and Kenya	a. Somewhat different “diagonal” interventions were implemented over 18 months in each site with four shared components: facilitating access to general health facilities, targeted peer outreach, targeted clinical services, and female sex worker empowerment. b. Before-and-after study design; each participant is their own control.	Follow-up: Unclear Uptake: 28.2% difference in “ever screened” from pre- to post-intervention (31.8 to 60.0%, *p* = 0.001) in South Africa; difference of 25.2% (0 to 25.2%, p = 0.001) in Mozambique; non-significant increase in Kenya (18.1 to 25.5%, *p* = 0.347).
Modibbo 2017 [27]	Women, aged 30–65, in semi-urban Nigeria	a. Health education on cervical cancer and its risk factors + self-sampling kit for at-home collection of HPV samples, to be mailed in or dropped-off at designated collection points. b. Same health education + an appointment for screening at a designated clinic.	Follow-up: 1 month after last enrollment Uptake: 91% difference in “ever screened” from pre- to post-intervention (1.5 to 92.5%) in intervention vs. 55.5% increase (1 to 56.5%) in control (p < 0.001).
Moses 2015 [28]	Women, aged 30–65, in Uganda	a. Outreach workers collected HPV specimens from women either in their home or a private area in their place of work using self-collection kits, transported samples to the laboratory each day, and shared results with participants by phone. b. Outreach workers gave women an appointment for VIA screening at the local health facility. Phone call reminders were placed 1 day before the appointment.	Follow-up: Unclear Uptake: 50.8% difference between intervention and control groups (99.2% vs. 48.4%, p < 0.001). Of the HR-HPV-positive women referred to the clinic for follow-up VIA testing, 45.2% attended the appointment.

^a^Intervention(s)

^b^Comparison(s)

### Effectiveness of interventions: screening uptake

Uptake of cervical screening was most commonly measured as “ever screened” (*n* = 8, 42.1%) or screened in a specified amount of time, usually during the study period (*n* = 7, 36.8%). One study created a screening score that accounted for presence of screening behavior, as well as when screening was last received and whether or not a woman was up-to-date with screening recommendations [19]. Some studies used vague language to describe uptake of cervical screening, which made it impossible to clearly deduce the measure of screening [16, 22, 28, 29, 32]. At baseline, screening coverage ranged from 0 to 53.6%, with most studies reporting coverage of less than 10% (*n* = 12, 63.2%). Following intervention, coverage ranged from 1.7 to 99.2%, with six studies reporting a significant improvement in screening that achieved ≥60% coverage (31.6%). More than a third of the studies reported non-significant increases in screening uptake associated with their intervention (*n* = 7, 36.8%); all of them were educational interventions [16, 17, 21, 29, 31–33].

One educational intervention was particularly effective, with a 64.4 percentage point increase in “ever screened” from 3.2% pre-intervention to 67.6% screened post-intervention (*p* < 0.001) [18]. This study evaluated a house-to-house education program that provided women with information about cervical and breast cancer and offered free screening services at health facilities within walking distance as well as HPV vaccination for eligible children for a fee. Two other educational interventions resulted in more modest, but significant improvements in coverage. One was an SMS behavior change communication intervention, offered with and without additional eVouchers to cover transportation costs to the screening clinic. Women who received the 15 SMS messages over 21 days had three times greater odds of screening during the study period than women in the control group (12.9% uptake vs. 4.3%, adjusted odds ratio [AOR] = 3.0, 95% CI: 1.5–6.2) [20]. Women randomized to receive the eVoucher for transportation in addition to the behavior change communication had almost five times the odds of uptake compared to the control group (18% uptake vs. 4.3%, AOR = 4.7, 95% CI: 2.93–7.44) [20]. Mbachu et al. designed a health education intervention that trained clergy wives in Anglican dioceses to deliver peer health education on cervical cancer to women they worship with. Women attending at least three group sessions reported a 6.8 percentage point increase in “ever screened” from 10.5% pre-intervention to 17.3% post-intervention (*p* = 0.02) [25].

As a category, the interventions that used innovative approaches to cervical cancer service delivery provided the most compelling results. A pilot RCT with 500 women in Uganda found that use of outreach workers to distribute and collect HPV self-sampling kits from women in their homes and workplaces was highly effective; 99.2% of self-collection samples were returned for HPV testing [28]. The control group, which received outreach worker visits, appointments for VIA screening at the local health facility, and phone call reminders the day before the appointment, also had good uptake (48.4%) [28]. The use of outreach workers was an effective screening implementation strategy and the use of self-sampling as the intervention further increased the uptake of screening behavior. Another study of home-based HPV self-sampling in Nigeria resulted in similar outcomes with 92.5% uptake in the intervention group post-intervention and 56.5% uptake in the control group post-intervention, compared to 1.5 and 1% uptake respectively in each group pre-intervention [27]. One study in rural Kenya offered community-based HPV screening at pop-up tents during a two-week phase of a six-week community health campaign. Women who screened on-the-spot in the tents had 60% uptake compared to the control community where women were referred to their local government health facility for testing, resulting in 37% uptake [23]. Another approach was to offer cervical screening as an add-on service for patients and their partners who were already seeking care in a facility. In a large RCT in urban Zambia, Hewett et al. offered enhanced client-centered counseling, referral to add-on services, and follow-up by phone if the appointment was missed [22]. Additionally, a second intervention group was provided escort services from the original service to the referral service location. A significant increase in the uptake of cervical screening was observed for both intervention arms compared to standard of care (without escort: 22.2% vs. 9.7%, AOR 2.75, 95% CI: 1.94–3.91; with escort: 23.6% vs. 9.7%, AOR = 2.98, 95%CI: 2.10–4.22) [22]. Another integrated services intervention targeted female sex workers in South Africa, Mozambique, and Kenya [24]. Implementation looked different in each site with shared components of increasing access to general health facilities, targeted peer outreach, targeted clinical services, and female sex worker empowerment. Significant increases in uptake were observed in South Africa (31.8 to 60.0%, *p* = 0.001) and Mozambique (0 to 25.2%) [24]. Lastly, combining cervical screening with a school-based HPV vaccination program in South Africa increased screening among mothers to 68.9%, though the prevalence of “ever screened” was higher at baseline (53.6%) compared to other included studies [19]. Both self-sampling and facility-based screening were offered with this intervention; a more favorable change was observed for the self-collection group with 45.3% of the screening kits being returned for testing [19].

### Secondary outcomes

Almost half of the included studies assessed some outcome other than screening uptake (*n* = 9, 47.4%): six had some measure of awareness (31.6%) [15, 18, 21, 29, 32, 33], five measured knowledge (26.3%) [15, 16, 19, 29, 32], one included an attitude score [16], and four reported “willingness to screen” (21.1%) [15, 25, 29, 33]. No studies used a validated measure for any of the secondary outcomes. Generally, improvements in secondary outcomes were observed, even when cervical screening did not significantly improve (Table 2). Willingness to screen was high before and after interventions, ranging from 63.4 to 89.7% of participants at baseline to 80.8 to 92.3% at follow-up.

### Theory of cervical screening behavior change

Theoretical frameworks were cited by several of the included studies, including the Health Belief Model [20], Pender’s Health Promotion Model [29], Behavior Change Theory [32], Prospect Theory [17], Diffusion of Innovation Theory [25], and the Intertemporal Choice Problem [26, 30]. We analyzed all included studies by applying the IBM and assessing whether IBM constructs were considered during intervention development, as the intervention mechanism, or as a measure/outcome of the intervention (Table 3). Knowledge and skills to perform the behavior was the construct considered most often (*n* = 13, 68.4%) followed by environmental constraints (*n* = 10, 52.6%). These constructs align with the intervention categories of educational interventions and innovative service delivery interventions, presented in Table 2. Economic incentivization interventions acted on the construct of instrumental attitude and operated under a premise that individuals must weigh the perceived costs/consequences and benefits of cervical screening and that behavior change can be prompted by increased immediate benefit or reduced cost of screening. Measures of instrumental attitude included individual risk assessments and perceived severity of cervical cancer. In addition to economic incentives, motivational interviewing was used to act on the construct of instrumental attitude, by helping patients to express and overcome individual barriers to screening and to improve self-efficacy [22]. Interventions employing peer or community health education acted on perceived norms (injunctive norms), in addition to providing knowledge to perform the behavior [18, 22, 24, 25]. Other strategies targeting norms included use of culturally-relevant media to disseminate information or demonstrations that modelled cervical cancer screening behavior [15, 16, 31].

**Table 3:** Number of studies addressing Integrated Behavior Model (IBM) constructs to increase uptake of cervical screening

	The IBM construct was considered				
IBM construct	During intervention development	As target of intervention	As a measure/ outcome	Total number of studies addressing construct N (%)	Studies and description of how construct was considered
Attitude					
Experiential	4	3	3	6 (31.6%)	Studies assessed attitudes and beliefs about cancer and screening and cited attitudes of health workers as a barrier to screening. Huchko 2018 [23], Mbachu 2017 [25], Modibbo 2017 [27], Moses 2015 [28], Okeke 2013 [30], Risi 2004 [31]
Instrumental	5	6	3	7 (36.8%)	Studies assessed perceptions of screening benefits, individual risk, and severity of cervical cancer. Motivational interviewing and incentivization interventions were used to overcome perceived barriers or increase perceived benefit. Adamu 2012 [15], Adonis 2014 [17], Hewett 2016 [22], Mbachu 2017 [25], Mehrotra 2014 [26], Okeke 2013 [30], Rosser 2015 [32]
Perceived norm					
Injunctive	2	4	0	4 (21.1%)	Community health workers, peer educators, and personal escorts used to social influence to promote positive attitudes of screening. Chigbu 2017 [18], Hewett 2016 [22], Lafort 2018 [24], Mbachu 2017 [25]
Descriptive	1	3	1	4 (21.1%)	Culturally-relevant media and peer programs modeled screening behavior. Abiodun 2014 [15], Huchko 2018 [23], Lafort 2018 [24], Risi 2004 [31]
Personal agency					
Perceived control	0	0	5	5 (26.3%)	Barriers to screening were assessed. Abiodun 2014 [15], Adamu 2012 [16], Mbachu 2017 [25], Ndikom 2017 [29], Wright 2011 [33]
Self-efficacy	2	2	0	2 (10.5%)	Motivational interviewing and screening demonstrations gave women confidence in their ability to screen and overcome identified barriers. Adamu 2012 [16], Hewett 2016 [22]
Knowledge and skills to perform	8	12	6	13 (68.4%)	Poor knowledge/awareness was cited as a major barrier to screening. Interventions used education to increase knowledge of cervical cancer, screening, and availability of screening services. Abiodun 2014 [15], Adamu 2012 [16], Adonis 2017 [17], Chigbu 2017 [18], Dreyer 2015 [19], Erwin 2019 [20], Gana 2017 [21], Mbachu 2017 [25], Ndikom 2017 [29], Okeke 2013 [30], Risi 2004 31], Rosser 2015 [32], Wright 2011 [33]
Environmental constraints	7	10	2	10 (52.6%)	Availability and accessibility of services were enhanced with free screening, transportation vouchers, and community-based screening. Studies measured type of transportation and distance to facility. Adamu 2012 [16], Dreyer 2015 [19], Erwin 2019 [20], Hewett 2016 [22], Huchko 2018 [23], Lafort 2018 [24], Modibbo 2017 [27], Moses 2015 [28], Okeke 2013 [30], Risi 2004 [31]
Habit	0	0	0	0 (0%)	Studies did not address habitual screening behavior.
Salience of behavior	0	0	2	2 (10.5)	Studies asked women if they would test again in the future and what their future screening preferences were. Huchko 2018 [23], Modibbo 2017 [27]
Intention to perform	1	0	4	4 (21.1%)	Intention was measured as “willingness to screen” among participants. Abiodun 2014 15], Mbachu 2017 [25], Ndikom 2017 [29], Wright 2011 [33]

Studies assessed experiential attitudes like fears and misconceptions about cervical screening when developing interventions that would be contextually-appropriate to their populations, and measured experiential attitude following interventions through acceptability questions like “was screening straight-forward/comfortable/convenient?” [23, 27]. Participants reported that screening methods were acceptable, indicating the experiential attitudes toward cervical cancer screening were positive overall. They would also recommend screening to friends, indicating positive descriptive norms (99.4 and 99.0% of self-sampling intervention arms) [23]. Salience of behavior could not be measured for women receiving screening for the first time but agreement with the statement that they would screen again in the future and future screening preferences indicate support for salience [23, 27]. Perceived control, a construct of personal agency, was measured by five studies that asked participants to identify barriers to screening [15, 16, 25, 29, 33]. Lack of awareness of screening services was unanimously the greatest perceived barrier to screening pre-intervention or in the control group among the five studies that measured participants’ perceived control and ranged from 41.4 to 94% [15, 16, 25, 29, 30]. For women participating in interventions, there was a shift following the intervention to citing other reasons for not screening which included lack of access or availability of screening services [15, 29], no symptoms/low perceived risk [25, 33], or not liking the test (Pap smear) [15]. Intention to perform the behavior or “willingness to screen” was measured by four of the studies (21.1%). Intention was high, even prior to intervention, with all four studies reporting that more than 70% of women were hypothetically willing to screen [15, 25, 29, 33].

## Discussion

Educational interventions were the most common type of intervention used to increase uptake of cervical screening in sub-Saharan Africa. The rationale for educational interventions is consistent with previous literature that names lack of knowledge or awareness as the most common barrier to cervical screening in low-and-middle-income countries and another review that found educational strategies were the most common among all cancer prevention efforts in sub-Saharan Africa [34, 35]. Educational interventions were not very effective overall. However, the studies that utilized peer health educators or community health educators as part of the implementation strategy were an exception [18, 25]. One of these studies provided one-time house-to-house education while the other required continued, on-going group engagement in a religious community setting. Both emphasized the role of social ties, using educators that were already known to the study participants. The SMS behavior change communication intervention also had modest but significant improvements in screening during the study period, with and without eVouchers provided for transportation, demonstrating that mHealth interventions may provide opportunity for future efforts to improve screening uptake especially as mobile phone ownership continues to grow, and that such approaches can be improved by adding interpersonal elements [20]. Invitations to screen and use of lay health advisors are two strategies that have been previously demonstrated to improve cervical screening behavior outside of SSA [36–38]. Educational interventions may also work when they are intensive, culturally-appropriate, based on health behavior models, and multi-dimensional so that they help women to overcome environmental constraints [36, 38]. Interventions that were not effective were short-term or one-time educational interventions, use of a photo-comic to deliver education, and framing of email messages.

Economic incentivization interventions were moderately effective, increasing uptake but still achieving less than 20% coverage [26, 30]. With only two studies incentivizing screening, there is also a paucity of evidence for this intervention type. Removing costs and increasing women’s perceived benefits of screening does lead to increased uptake of screening, but the low post-intervention coverage indicates that further action is needed to reach desirable coverage. This approach may be combined with other types of interventions in the future, like those that consider environmental constraints and act on instrumental attitude simultaneously. Several studies did include eVouchers to cover transportation costs, or purposely selected communities where screening was available within walking distance, which may be a best practice. This claim is supported by the finding that a 10-min increased travel time to the clinic reduced participation by 1.5 percentage points in one study [30], that “distance to the screening center” was identified as a barrier to screening post-intervention by 16.2 and 21.6% of women in two other studies [25, 29], and that eVouchers for transportation, when added to an SMS messaging intervention, increased odds of screening compared to the intervention without eVouchers for transportation (AOR: 1.53, 95% CI: 1.11–2.19) [20].

Innovative service delivery interventions worked by changing the location of screening services, or by combining screening services with other health services such as voluntary male circumcision and HPV vaccination. The six studies in our review that were categorized as innovative service delivery approaches focus on the availability, accessibility, and appropriateness of screening services for women, acting on the IBM construct of environmental constraints. Categorically, these interventions resulted in the greatest increases in screening uptake.

### Appropriateness of IBM to explain cervical screening

The IBM operates on the premise that behavior is most influenced by the intent to perform that behavior. In this review, we observed very high willingness to screen, even pre-intervention, but that intent was not always well-translated to uptake of cervical cancer screening. Ndikom et al. described an increase from 75.8 to 91.0% in willingness to screen yet no change in actual screening [29]. Furthermore, one of the included studies saw a significant improvement in uptake without any significant change in willingness to screen [25]. Through application of the health behavior model, we can begin to examine how interventions may work and explore other factors that may be disrupting the translation of behavioral intent to behavior. For example, intention may not translate to desired behavior when knowledge or skills to perform the behavior are low or when environmental constraints prevent a person from following through on the intent. Two educational interventions found that after intervention, participants were less likely to identify knowledge or awareness as a barrier to screening but more likely to identify non-availability of services [15, 29]. The reality is that low awareness and availability issues probably need to be addressed concurrently. We suggest more research into health systems change interventions that address infrastructure and human resource challenges, patient-centered healthcare delivery, and reducing “visit burden” for patients and facilities. While these types of interventions act on the environmental constraints construct of the IBM, another theoretical framework may be more appropriate for systems change interventions where individual behavioral intention is not the major determinant of behavior change.

Habit related to the behavior of cervical screening was not considered by any of the studies, likely because the goal of most interventions was to get women to screen for the first time rather than to instill a new habitual health behavior. Two studies assessed intended salience of behavior by asking participants if they would test again in the future and what their preferences would be [23, 27]. Very few interventions considered the element of self-efficacy, perhaps in part because of the focus on environmental constraints beyond a patient’s control, rather than internal factors. Techniques like motivational interviewing, in combination with the removal of environmental constraints may be one way to increase women’s personal agency and should be explored through future research as a method of improving uptake.

### Interventions that target male partners and families as an opportunity

One included study involved males, by offering referral services to patients and their partners seeking care at facilities in the study catchment area [22]. This intervention offered cervical screening as an add-on service for all women seeking care and for female partners of all men seeking care. Male partners of females seeking care were also referred for services like HIV testing and counseling and voluntary medical male circumcision. During the intervention development phase, Risi et al. found that men, particularly father-in-laws and husbands, played an important role in decision-making related to Pap smear uptake in South Africa [31]. Finding ways to meaningfully engage with male partners and relatives may therefore be an effective approach to increasing cervical screening in women. A review of barriers to cervical cancer screening in low-and-middle income countries found that lack of family support was the most commonly reported sociocultural/religious barrier [34]. Indeed, control group participants in one of our included studies, set in Ibadan, Nigeria, reported lack of support from husband (35%) and lack of decision-making ability (37%) as barriers to screening [29]. When executed strategically and sensitively, positive male partner engagement has proven valuable for promotion of other health behaviors such as adoption of HIV prevention behavior following couples-based counseling, testing, and services [39–41]. Male partner involvement has also shown to effect cervical cancer-related behavior change; in Uganda, male partner involvement reduced loss-to-follow-up among women referred for colposcopy [42].

### Screening methods

In this review, we considered uptake of any type of cervical screening as the behavior of interest, but a question remains as to whether screening with different methodologies is one behavior or multiple behaviors. Is the behavior of screening with Pap smear truly the same as accepting visual inspection with acetic acid (VIA) or HPV testing? While some behavioral change constructs, like knowledge of cervical cancer, are shared between screening methodologies, attitudes toward each screening method may differ. For example, Adamu et al. found that 38.4% of their intervention group did “not like Pap smear” post-intervention [16]. Interestingly, this figure was much higher than at baseline when only 6.7% of the intervention group and 1.3% of the control group indicated a dislike for the test. In contrast, HPV self-sampling interventions in Nigeria and Uganda where more than 90% of self-sample kits were returned for testing were very favorably responded to by participants not only in terms of screening behavior but also in self-reported attitudes toward the screening procedure [27, 28]. When asked, 83.2% of women in one study indicated a preference for self-sampling in the future, while 9.2% preferred hospital-sampling, and 7.6% had no preference [27]. Among the women with a preference for self-sampling, they liked the test’s comfort (87%), privacy (6.5%), and found it to be less embarrassing (6.5%) [27]. To women, the act of collecting a cervicovaginal swab in the comfort of their own home may be conceptualized very differently than presenting on an exam table and exposing themselves to a health worker for a cervical exam. Future investigation into women’s conceptualization of each type of screening may inform behavior change interventions targeting each type of screening. Additionally, we recommend transparency of screening methods in reporting; one limitation we encountered was an inability to deduce the type of screening in five of the included studies (26.3%) [16, 22, 28, 29, 32].

In this review, screening uptake was highest among all screening methods for HPV testing of self-collected samples. Screen-and-treat recommendations favor use of HPV testing either alone or in sequence with other screening methods when feasible to identify high-risk, HPV-positive women [10]. Of the four studies in this review that used HPV testing, two studies referred HPV-positive women for VIA as a sequential screening method [28] or to determine treatment eligibility [23], while the remaining studies vaguely described follow-up that included inviting HPV-positive women for further investigation and treatment if necessary [19, 27]. One advantage of using HPV testing is that it frees up the screening program staff to spend more time with high-risk patients by triaging out the HPV-negative women. This screening method may, however, be more prone to loss-to-follow-up (LFU) than visual inspection screening methods. In particular, the out-of-facility location of testing possible with the method, the possible extra step added to a screen-and-treat approach, and test results that are not immediately available to health workers and women create opportunity for LFU. The endpoint or outcome of public health significance may therefore not be just the proportion of women who return a sample, but those who are notified and counseled on their results, and the number of women who go on to receive subsequent complementary cervical screening or treatment. Moses et al. utilized home- and workplace-based HPV self-sampling but found that 53.4% of the HPV-positive women in their sample were unable to be reached by phone after three attempts by the community outreach worker and ultimately 45.2% attended a follow-up VIA appointment at the health center [28]. Huchko et al. observed low treatment acquisition of HPV-positive women identified through their community health campaigns (39.2%) but observed an even lower treatment acquisition rate among women who were tested for HPV in facilities (31.5%), demonstrating that the challenge isn’t necessarily about getting women into the clinic [23]. They describe a long time between screening and treatment, 47 days on average with no difference between the study arms, as one area for LFU improvement. Other recommendations include improved follow-up protocols, use of varied result notification methods like text or in-person home-visits, and having women program the caller’s phone number into their phone at time of sample collection so that they will recognize the caller’s number later at time of notification [28]. The “screen-and-treat” approach common in Africa keeps LFU at a minimum by performing the screening, sharing the results, and treating any possible pre-cancerous lesions during the same health visit. Approaches that use HPV testing should be especially aware of LFU and proactively work to mitigate its effects.

### Urban/rural divide

This review highlights greater gains in screening uptake following intervention in rural communities compared with urban study settings. Community awareness about cervical cancer and screening and access to screening may be lower in rural communities at baseline. One study found that only 10% of rural women had heard of cervical screening before and “good knowledge” increased from 2 to 70.5% following intervention [15]. The intervention by Chigbu et al., that increased screening from 3.2 to 67.6%, noted that of the women who were screened following intervention, 94.3% were not aware of cervical screening before the intervention [18]. One study included in this review operated in both urban and rural locations and further demonstrates the urban/rural difference in screening; participants in the SMS behavior change communication intervention with eVoucher for transportation had a more pronounced effect in the rural community than urban community (AOR: 8.78 vs. AOR: 4.67) [20]. Dramatic results in communities previously unexposed to information about screening and with underdeveloped health services may not be indicative of sustained increases in uptake or comparable to what is possible for urban sites. Still, screening even just once in a lifetime is beneficial and the WHO recommends increasing population coverage over increasing the number of times an individual woman is screened [10].

This is the first study, to our knowledge, to review literature on interventions to increase uptake of cervical screening in sub-Saharan Africa, a region with cervical cancer epidemiology distinct from other world regions and from other low-and-middle income countries. We apply a health behavior model to understand how interventions might work and identify opportunities for future research on cervical screening. Rigorous methodology for systematic searching, screening, and data charting and use of PRISMA-ScR reporting maximizes the review’s reproducibility.

### Limitations

One limitation of this review is that 14 of 19 studies come from just two countries, Nigeria and South Africa. While we are attempting to draw conclusions at a regional level for sub-Saharan Africa, the lack of diversity in countries may limit the generalizability of findings. Still, a variety of settings and populations were represented from studies within those two countries and one study compared “diagonal” interventions across countries, juxtaposing results from South Africa to those from Mozambique and Kenya [24]. Careful construction of a search strategy that utilized individual country names for all SSA countries mitigated the possibility that South African studies would be returned more often than other countries for search terms like “Africa”. Rather, we believe that studies from these two countries are just more present in the literature. As a U.S.-based research team, we also acknowledge potential bias in the literature sources searched which may not fully capture the breadth of African literature. Missed citations could exist in Africa-specific databases or journals that we did not hand-search.

Our search may have an over-representation of Pap smear screening because of the study designs and locations of included studies. Pap smear may be seen as a gold standard for screening and may therefore be employed by RCTs and quasi-experimental studies. Pap smears may also be more widely available in certain countries like South Africa. Another review of cervical cancer prevention in sub-Saharan Africa found that VIA was the most commonly used secondary screening method [35]. Both VIA and HPV testing have been declared strategies for closing the cancer divide between LMIC and high-income countries because of their cost-effectiveness and feasibility [43] so the over-representation of Pap smear testing in our study may limit the generalizability and significance of our findings. Lastly, heterogeneity of screening methods, screening uptake measures, and lack of validated measures for secondary outcomes made it difficult to compare results across studies. Work to validate cervical cancer awareness, knowledge, attitude, belief, and personal agency measures should be undertaken in sub-Saharan Africa.

## Conclusions

While educational interventions acting on one’s knowledge and skills to perform the behavior of cervical screening were the most common type of interventions identified in our review, they were minimally effective. The exception were interventions utilizing peer educators, mHealth interventions, and strategies that acted on multiple constructs from the IBM like education and environmental constraints simultaneously. Innovative approaches to cervical cancer screening service delivery, including community-based HPV self-sampling, demonstrated promising changes to uptake in screening. However, a different type of theoretical framework may be better suited to evaluate health systems change interventions. The IBM’s central premise, that intention to perform behavior is the major determinant of behavior change, was not supported by the studies in our review. We call for improved reporting of screening methods and screening outcomes including “willingness to screen” by other researchers. We also suggest further investigation into strategies that engage male partners and family members, and measurement and manipulation of personal agency to overcome barriers to screening.

## Supplementary information

**Additional file 1.** Appendix 1. Complete systematic searches conducted on May 1st, 2019. This appendix presents the comprehensive search strategy for all electronic databases searched (PubMed, Web of Science, Embase, CINAHL) with controlled vocabulary and key terms, as well as the number of search results returned.

**Additional file 2.** Lott_PRISMA-ScR Checklist. Preferred Reporting Items for Systematic reviews and Meta-Analyses extension for Scoping Reviews (PRISMA-ScR) Checklist. Completed checklist for the scoping review, using the PRISMA Extension for Scoping Reviews.

## Data Availability

Not applicable.

## Abbreviations

IBM: Integrated Behavioral Model
HIV: Human Immunodeficiency Virus
HPV: Human papillomavirus
LMIC: Low-and-middle-income countries
LFU: Loss-to-follow-up
PICO: Population, intervention, comparison, outcome
PRISMA-ScR: Preferred Reporting Items for Systematic reviews and Meta-Analyses extension for Scoping Reviews
RCT: Randomized controlled trial
SSA: Sub-Saharan Africa
VIA: Visual inspection with acetic acid
VILI: Visual inspection with Lugol’s iodine
