# Supplementary material for: Interventions to increase uptake of cervical screening in sub-Saharan Africa: a scoping review using the integrated behavioral model
Source: BMC Public Health. 2020 May 11;20:654. doi: 10.1186/s12889-020-08777-4 (PMC7216595; doi:10.1186/s12889-020-08777-4)
Supplement: Supplementary file 1 — Additional file 1. Appendix 1. Complete systematic searches conducted on May 1st, 2019. This appendix presents the comprehensive search strategy for all electronic databases searched (PubMed, Web of Science, Embase, CINAHL) with controlled vocabulary and key terms, as well as the number of search results returned. [file 12889_2020_8777_MOESM1_ESM.docx]

**Appendix 1. Complete systematic searches conducted on May 1^st^, 2019**

| **Database** | **Search**  (Cancer Terms) AND (Screening/Outcome Terms) AND (Context Terms) | **Search Results** |
| --- | --- | --- |
|  |  |  |
| PubMed | ("Uterine Cervical Neoplasms/prevention and control"[MESH] OR "Uterine Cervical Neoplasms/diagnosis"[MESH] OR "Uterine Cervical Dysplasia/prevention and control"[MESH] OR "Uterine Cervical Dysplasia/diagnosis"[MESH] OR "Cervical Intraepithelial Neoplasia"[MESH] OR "Cervical neoplasm" OR "Cervical neoplasms" OR "Uterine cervical neoplasm" OR "Uterine cervical neoplasms" OR "Cervix neoplasms" OR "Cervix neoplasm" OR "Cancer of the uterine cervix" OR "Cancer of the cervix" OR "Cervical cancer" OR "Cervical cancers" OR "Uterine cervical cancer" OR "Uterine cervical cancers" OR "Cervix cancer" OR "Cervix cancers" OR "Cervical dysplasia" OR "Uterine cervical dysplasia" OR "Cervix dysplasia")  AND  ("Early Detection of Cancer"[MESH] OR "Mass Screening"[MESH] OR "Papillomavirus Infections/diagnosis"[MESH] OR "Papanicolaou Test"[MESH] OR "Human Papillomavirus DNA Tests"[MESH] OR "Acetic Acid"[MESH] OR "Iodides"[MESH] OR "Vaginal Smears"[MESH] OR "Cancer early detection" OR "Cervical cancer screening" OR "Cancer screening" OR "Early diagnosis of cancer" OR "Cancer early diagnosis" OR “Screen-and-treat” OR "Screening" OR "Visual inspection" OR "Visual method" OR "Visual methods" OR "Acetic acid" OR "Lugol's" OR "VIA/VILI" OR "HPV test" OR "HPV tests" OR "HPV testing" OR "HPV DNA test" OR "HPV DNA tests" OR "HPV DNA testing" OR "Human papillomavirus testing" OR "Human papillomavirus test" OR "Human papillomavirus tests" OR "Pap smear" OR "Pap smears" OR "Papanicolaou smear" OR "Pap test" OR "Pap testing" OR "Papanicolaou test" OR "Papanicolaou testing" OR "Vaginal smear" OR "Vaginal smears" OR "Cytology" OR "Smear cytology" OR "Vaginal cytology" OR "colposcopy")  AND  ("Africa"[MESH] OR "Africa" OR "African" OR "Africans" OR "Sub-Saharan Africa" OR "Sub-Saharan African" OR "Sub-Saharan Africans" OR "Cameroon" OR "Central African Republic" OR "Chad" OR "Congo" OR "Democratic Republic of the Congo" OR "Equatorial Guinea" OR "Gabon" OR "Sao Tome and Principe" OR "Sao Tome" OR "Burundi" OR "Djibouti" OR "Eritrea" OR "Ethiopia" OR "Kenya" OR "Rwanda" OR "Somalia" OR "South Sudan" OR "Sudan" OR "Tanzania" OR "Uganda" OR "Angola" OR "Botswana" OR "Lesotho" OR "Malawi" OR "Mozambique" OR "Namibia" OR "South Africa" OR "Swaziland" OR "Eswatini" OR "Zambia" OR "Zimbabwe" OR "Benin" OR "Burkina Faso" OR "Cabo Verde" OR "Cote d’Ivoire" OR "Ivory Coast" OR "Gambia" OR "Ghana" OR "Guinea" OR "Guinea-Bissau" OR "Liberia" OR "Mali" OR "Mauritania" OR "Niger" OR "Nigeria" OR "Senegal" OR "Sierra Leone" OR "Togo" OR "Madagascar") | 1,867 |
| Embase | ('uterine cervix cancer'/exp OR 'uterine cervix cancer' OR 'uterine cervix dysplasia'/exp OR 'uterine cervix dysplasia' OR 'cervical neoplasm' OR 'cervical neoplasms' OR 'uterine cervical neoplasm' OR 'uterine cervical neoplasms'/exp OR 'uterine cervical neoplasms' OR 'cervix neoplasm' OR 'cervix neoplasms'/exp OR 'cervix neoplasms' OR 'cancer of the uterine cervix' OR 'cancer of the cervix' OR 'cervical cancer'/exp OR 'cervical cancer' OR 'cervical cancers' OR 'uterine cervical cancer'/exp OR 'uterine cervical cancer' OR 'uterine cervical cancers' OR 'cervix cancer'/exp OR 'cervix cancer' OR 'cervix cancers' OR 'cervical dysplasia'/exp OR 'cervical dysplasia' OR 'uterine cervical dysplasia'/exp OR 'uterine cervical dysplasia' OR 'cervix dysplasia'/exp OR 'cervix dysplasia')  AND  ('early cancer diagnosis'/exp OR 'early cancer diagnosis' OR 'mass screening'/exp OR 'mass screening' OR 'human papillomavirus dna test'/exp OR 'human papillomavirus dna test' OR 'iodide'/exp OR 'iodide' OR 'cancer early detection' OR 'cervical cancer screening' OR 'cancer screening'/exp OR 'cancer screening' OR 'early diagnosis of cancer' OR 'cancer early diagnosis' OR 'screen-and-treat' OR 'screening'/exp OR 'screening' OR 'visual inspection'/exp OR 'visual inspection' OR 'visual method' OR 'visual methods' OR 'acetic acid'/exp OR 'acetic acid' OR 'lugols' OR 'via/vili' OR 'hpv test*'/exp OR 'hpv test*' OR 'hpv dna test*' OR 'human papillomavirus test*'/exp OR 'human papillomavirus test*' OR 'pap smear*'/exp OR 'pap smear*' OR 'papanicolaou smear'/exp OR 'papanicolaou smear' OR 'pap test*'/exp OR 'pap test*' OR 'papanicolaou test*'/exp OR 'papanicolaou test*' OR 'vaginal smear*'/exp OR 'vaginal smear*' OR 'cytology'/exp OR 'cytology' OR 'smear cytology' OR 'vaginal cytology'/exp OR 'vaginal cytology' OR 'colposcopy'/exp OR 'colposcopy')  AND  ('africa'/exp OR 'africa' OR 'african'/exp OR 'african' OR 'africans'/exp OR 'africans' OR 'sub-saharan africa'/exp OR 'sub-saharan africa' OR 'sub-saharan african' OR 'sub-saharan africans' OR 'cameroon'/exp OR 'cameroon' OR 'central african republic'/exp OR 'central african republic' OR 'chad'/exp OR 'chad' OR 'congo'/exp OR 'congo' OR 'democratic republic of the congo'/exp OR 'democratic republic of the congo' OR 'equatorial guinea'/exp OR 'equatorial guinea' OR 'gabon'/exp OR 'gabon' OR 'sao tome and principe'/exp OR 'sao tome and principe' OR 'sao tome' OR 'burundi'/exp OR 'burundi' OR 'djibouti'/exp OR 'djibouti' OR 'eritrea'/exp OR 'eritrea' OR 'ethiopia'/exp OR 'ethiopia' OR 'kenya'/exp OR 'kenya' OR 'rwanda'/exp OR 'rwanda' OR 'somalia'/exp OR 'somalia' OR 'south sudan'/exp OR 'south sudan' OR 'sudan'/exp OR 'sudan' OR 'tanzania'/exp OR 'tanzania' OR 'uganda'/exp OR 'uganda' OR 'angola'/exp OR 'angola' OR 'botswana'/exp OR 'botswana' OR 'lesotho'/exp OR 'lesotho' OR 'malawi'/exp OR 'malawi' OR 'mozambique'/exp OR 'mozambique' OR 'namibia'/exp OR 'namibia' OR 'south africa'/exp OR 'south africa' OR 'swaziland'/exp OR 'swaziland' OR 'eswatini' OR 'zambia'/exp OR 'zambia' OR 'zimbabwe'/exp OR 'zimbabwe' OR 'benin'/exp OR 'benin' OR 'burkina faso'/exp OR 'burkina faso' OR 'cabo verde'/exp OR 'cabo verde' OR 'cote d ivoire' OR 'ivory coast'/exp OR 'ivory coast' OR 'gambia'/exp OR 'gambia' OR 'ghana'/exp OR 'ghana' OR 'guinea'/exp OR 'guinea' OR 'guinea-bissau'/exp OR 'guinea-bissau' OR 'liberia'/exp OR 'liberia' OR 'mali'/exp OR 'mali' OR 'mauritania'/exp OR 'mauritania' OR 'niger'/exp OR 'niger' OR 'nigeria'/exp OR 'nigeria' OR 'senegal'/exp OR 'senegal' OR 'sierra leone'/exp OR 'sierra leone' OR 'togo'/exp OR 'togo' OR 'madagascar'/exp OR 'madagascar') | 3,013 |
| Web of Science | TS=("Uterine cervix cancer" OR "Uterine cervix dysplasia" OR "Cervical neoplasm" OR "Cervical neoplasms" OR "Uterine cervical neoplasm" OR "Uterine cervical neoplasms" OR "Cervix neoplasms" OR "Cervix neoplasm" OR "Cancer of the uterine cervix" OR "Cancer of the cervix" OR "Cervical cancer" OR "Cervical cancers" OR "Uterine cervical cancer" OR "Uterine cervical cancers" OR "Cervix cancer" OR "Cervix cancer" OR "Cervical dysplasia" OR "Uterine cervical dysplasia" OR "Cervix dysplasia")  AND  TS=("Early cancer diagnosis" OR "Mass Screening" OR "Papanicolaou Test" OR "Human Papillomavirus DNA Test" OR "Acetic Acid" OR "Iodide" OR "Vaginal Smear" OR "Cancer early detection" OR "Cervical cancer screening" OR "Cancer screening" OR "Early diagnosis of cancer" OR "Cancer early diagnosis" OR "Screen-and-treat" OR "Screening" OR "Visual inspection" OR "Visual method" OR "Visual methods" OR "Acetic acid" OR "Lugols" OR "VIA/VILI" OR "HPV test" OR "HPV tests" OR "HPV testing" OR "HPV DNA test" OR "HPV DNA tests" OR "HPV DNA testing" OR "Human papillomavirus testing" OR "Human papillomavirus test" OR "Human papillomavirus tests" OR "Pap smear" OR "Pap smears" OR "Papanicolaou smear" OR "Pap test" OR "Pap testing" OR "Papanicolaou test" OR "Papanicolaou testing" OR "Vaginal smear" OR "Vaginal smears" OR "Cytology" OR "Smear cytology" OR "Vaginal cytology" OR "Colposcopy")  AND  TS=("Africa" OR "African" OR "Africans" OR "sub-Saharan Africa" OR "sub-Saharan African" OR "sub-Saharan Africans" OR "Cameroon" OR "Central African Republic" OR "Chad" OR "Congo" OR "Democratic Republic of the Congo" OR "Equatorial Guinea" OR "Gabon" OR "Sao Tome and Principe" OR "Sao Tome" OR "Burundi" OR "Djibouti" OR "Eritrea" OR "Ethiopia" OR "Kenya" OR "Rwanda" OR "Somalia" OR "South Sudan" OR "Sudan" OR "Tanzania" OR "Uganda" OR "Angola" OR "Botswana" OR "Lesotho" OR "Malawi" OR "Mozambique" OR "Namibia" OR "South Africa" OR "Swaziland" OR "Eswatini" OR "Zambia" OR "Zimbabwe" OR "Benin" OR "Burkina Faso" OR "Cabo Verde" OR "Cote dIvoire" OR "Ivory Coast" OR "Gambia" OR "Ghana" OR "Guinea" OR "Guinea-Bissau" OR "Liberia" OR "Mali" OR "Mauritania" OR "Niger" OR "Nigeria" OR "Senegal" OR "Sierra Leone" OR "Togo" OR "Madagascar") | 1,436 |
| CINAHL | (MH "Cervical intraepithelial Neoplasia" OR MH "Uterine Neoplasms+" OR MH "Cervix dysplasia" OR "Cervical neoplasm" OR "Cervical neoplasms" OR "Uterine cervical neoplasm" OR "Uterine cervical neoplasms" OR "Cervix neoplasms" OR "Cervix neoplasm" OR "Cancer of the uterine cervix" OR "Cancer of the cervix" OR "Cervical cancer" OR "Cervical cancers" OR "Uterine cervical cancer" OR "Uterine cervical cancers" OR "Cervix cancer" OR "Cervix cancers" OR "Cervical dysplasia" OR "Uterine cervical dysplasia" OR "Cervix dysplasia")  AND  (MH "Cervical Smears+" OR MH "Colposcopy" OR MH "Cancer screening" OR MH "Early Detection of Cancer" OR MH "Acetic Acid" OR MH "Iodine" OR MH "Diagnostic tests, routine" OR "Cancer early detection" OR "Cervical cancer screening" OR "Cancer screening" OR "Early diagnosis of cancer" OR "Cancer early diagnosis" OR “Screen-and-treat” OR "Screening" OR "Visual inspection" OR "Visual method" OR "Visual methods" OR "Acetic acid" OR "Lugol's" OR "VIA/VILI" OR "HPV test" OR "HPV tests" OR "HPV testing" OR "HPV DNA test" OR "HPV DNA tests" OR "HPV DNA testing" OR "Human papillomavirus testing" OR "Human papillomavirus test" OR "Human papillomavirus tests" OR "Pap smear" OR "Pap smears" OR "Papanicolaou smear" OR "Pap test" OR "Pap testing" OR "Papanicolaou Test" OR "Papanicolaou testing" OR "Vaginal smear" OR "Vaginal smears" OR "Cytology" OR "Smear cytology" OR "Vaginal cytology" OR "Colposcopy")  AND  (MH "Africa+" OR "Africa" OR "African" OR "Africans" OR "sub-Saharan Africa" OR "sub-Saharan African" OR "sub-Saharan Africans" OR "Cameroon" OR "Central African Republic" OR "Chad" OR "Congo" OR "Democratic Republic of the Congo" OR "Equatorial Guinea" OR "Gabon" OR "Sao Tome and Principe" OR "Sao Tome" OR "Burundi" OR "Djibouti" OR "Eritrea" OR "Ethiopia" OR "Kenya" OR "Rwanda" OR "Somalia" OR "South Sudan" OR "Sudan" OR "Tanzania" OR "Uganda" OR "Angola" OR "Botswana" OR "Lesotho" OR "Malawi" OR "Mozambique" OR "Namibia" OR "South Africa" OR "Swaziland" OR "Eswatini" OR "Zambia" OR "Zimbabwe" OR "Benin" OR "Burkina Faso" OR "Cabo Verde" OR "Cote d’Ivoire" OR "Ivory Coast" OR "Gambia" OR "Ghana" OR "Guinea" OR "Guinea-Bissau" OR "Liberia" OR "Mali" OR "Mauritania" OR "Niger" OR "Nigeria" OR "Senegal" OR "Sierra Leone" OR "Togo" OR "Madagascar") | 669 |
